# Influences of joint dynamics on take-off velocity in elite male 110-m hurdlers

**DOI:** 10.3389/fspor.2025.1733252

**Published:** 2026-10-01

**Authors:** Kerui Wang, Yuzhang Li

**Affiliations:** School of Physical Education, Shanghai University of Sport, Shanghai, China

**Keywords:** 110-m hurdles, joint moments, joint stiffness, kinematics, kinetics

## Abstract

**Objective:**

To study the relationship between lower limb joint moments, joint stiffness, and take-off velocity in high-level hurdlers.

**Methods:**

This study employed a Qualisys three-dimensional infrared motion-capture system synchronized with a force plate. Based on inverse dynamics principles, kinematic and kinetic data were collected during the clearance phase over the fourth hurdle in 16 elite male 110-m hurdlers.

**Results:**

During the landing phase, Group B had significantly higher peak joint moments at the hip, knee, and ankle compared to Group A (*P* < 0.01). During the propulsive phase, Group B had significantly higher peak joint moments at the hip, knee, and ankle compared to Group A(*P* < 0.01). Group B had significantly higher joint stiffness at the hip, knee, and ankle compared to Group A (*P* < 0.01). During the landing phase, the relative peak joint moments at the hip, knee, and ankle were positively correlated with landing velocity, with correlation coefficients (*r*) ranging from 0.51 to 0.61. During the propulsive phase, the relative peak moments at these joints were highly positively correlated with take-off velocity, with *r* values between 0.82 and 0.89. Hip, knee, and ankle joint stiffness was highly positively correlated with clearance velocity (*r*: 0.80–0.90) but highly negatively correlated with the rate of change in velocity (*r*: −0.88 to −0.95).

**Conclusions:**

During the take-off phase, Group B exhibited greater relative peak joint moments and joint stiffness at the hip, knee, and ankle compared to Group A. These measures were strongly correlated with clearance velocity, indicating that increasing joint moments and stiffness contributes to faster hurdle clearance. Among the lower-limb joints, hip and ankle performance had the most significant influence on clearance mechanics. Additionally, the fitted functional equations enable prediction of clearance velocity from joint moment data. Therefore, incorporating stretch-shortening cycle exercises in training to enhance lower-limb strength, increase joint moments, and improve joint stiffness should effectively enhance performance and competitive outcomes.

## Introduction

Hurdling is one of thfe most prominent events in the Olympic Games and major athletics competitions. The men's 110 m hurdles is a discipline that tightly integrates speed and technique, requiring athletes to clearance ten hurdles in the shortest possible time (1). Elite hurdlers must possess exceptional velocity, coordination, reactive strength, technical proficiency, as well as outstanding flexibility and balance (2–4). The hurdle step is divided into three phases: the take-off phase, the hurdle clearance phase, and the landing phase (initial and second contacts) (5). The take-off phase is the beginning of the hurdle clearance and serves as the critical transition from running to hurdling, the take-off phase was defined as the moment when the foot first contacted the ground until the toe-off. The take-off phase consists of the landing phase and the propulsive phase (1). The quality of the take-off directly affects the trajectory of the body's center of mass and hurdle clearance time, and during the take-off phase, while also influencing the stability of landing and the smooth integration with subsequent running strides (6–8). Studies suggest that strong support and extension capacities of the lower-limb joints are essential during the take-off phase. Previous studies have found that athletes typically reach their maximum speed at the fourth and fifth hurdles (9). Specifically, the speed pattern of hurdle runners varies across different hurdles, with the fourth hurdle often being the point of maximum speed (5). There are significant differences in the speed patterns between the fourth hurdle and other hurdles (10). Therefore, this study focuses on the fourth hurdle to investigate the biomechanical performance of athletes during the take-off phase.

Joint kinetics analysis holds considerable significance in hurdling research (11). Previous studies have examined ground reaction forces and impulse characteristics in hurdling (9, 12). However, research employing inverse dynamics to calculate joint moments and joint stiffness during hurdling remains limited. It is widely recognized that peak joint moments in the lower limbs are critical indicators of technical performance in high-speed movements and jumping activities, as they directly determine the athlete's force output capacity (13–16). Joint stiffness is defined as the capacity of a joint to resist deformation under loading (13). Evidence indicates that joint stiffness is associated with running speed and jump height (17, 18), enhancing joint stiffness is therefore of great importance for improving athletic performance and provides a scientific basis for training interventions (19–21). In competitive sports, high-intensity exercise is highly prone to inducing injuries (22), Joint stiffness may also be associated with sports injuries: excessive stiffness can lead to skeletal and muscular damage, whereas insufficient stiffness may increase the risk of soft tissue injury (23).

Many current studies have explored the technical differences between elite and sub-elite athletes, as well as between athletes of different genders. However, most of these studies primarily focus on the kinematic characteristics of the hurdle stride. A systematic review of the kinematics of different phases of the hurdle stride concluded that biomechanical analysis and technical improvements can enhance performance. However, the review indicates the need for further research, such as studies on the dynamics of hurdling, to better understand how hurdling technique affects performance (10, 24–26). Additionally, some studies have analyzed the Ground Reaction Force (GRF) during the fourth hurdle, revealing that higher vertical GRF and reduced negative force during the landing phase are necessary to achieve a higher center of mass (9). This study employed a three-dimensional motion capture system in combination with force platforms to investigate differences in lower-limb joint kinetics across athletes of varying competitive levels from the perspective of inverse dynamics. It further explored the relationship between joint kinetics and take-off velocity, aiming to clarify the specific contributions and underlying mechanisms of kinetic indicators to take-off performance. The objective of this study is to provide theoretical guidance and quantitative evidence for hurdling training, thereby enabling athletes to systematically enhance lower-limb strength, stiffness, and technical stability, ultimately advancing competitive performance in hurdling.

The study hypothesis suggests that elite athletes exhibit greater joint moment and joint stiffness in the hip, knee, and ankle. Moreover, joint moment and joint stiffness are correlated with take-off velocity, with the hip and ankle joints contributing more significantly than the knee joint in terms of lower limb biomechanics.

## Methods

### Participants

This study included 16 male hurdling athletes, grouped based on the criteria outlined in the International Journal of Sports Physiology and Performance (27). Eight subelite (Group A) and eight elite (Group B) athletes were recruited in this study. Group A was classified as tier 3, and Group B as tier 4 (Table 1).

**Table 1:** Athletes information.

Participants	Group A(tier 3)	Group B(tier 4)
	(*n* = 8)	(*n* = 8)
Age (yr)	22 ± 3	23 ± 1
Height (m)	1.90 ± 0.03	1.90 ± 0.04
Body Mass (kg)	81.25 ± 4.43	76.88 ± 2.80
Personal Best (s)	14.06 ± 0.11	13.52 ± 0.21*

* *P* < 0.05.

All athletes had no history of lower limb musculoskeletal injuries in the previous 6 months and refrained from strenuous exercise for 24 h before the study. Each participant signed an informed consent form before the experiment.

### Data collection

The experiment was conducted at the indoor track and field facility of Shanghai Second Sports School. Ten hurdles were positioned along the indoor straight track, spaced 9.14 m apart, each with a height of 1.067 m. The distance from the start line to the first hurdle was 13.72 m. Kinematics data were collected using a 10-camera infrared three-dimensional (3D) motion capture system (Qualisys2024, Oqus600, Sweden) at a sampling rate of 200 Hz and cutoff frequency of 10 Hz (28, 29). Sixty infrared retroreflective markers, each with a diameter of 15.0 mm, were attached bilaterally to the lower limbs to define the hip, knee, and ankle joints according to the plug-in gait marker set. Five Kistler 3D force platforms (9,287 C, 900 mm × 600 mm × 100 mm, Switzerland) were used, sampling at 2,000 Hz with a cutoff frequency of 50 Hz (9, 30), These platforms were consecutively placed at the fourth hurdle, as maximum hurdling speed typically occurs at this point (9). Initial foot contact and liftoff moments were determined using a 20 N threshold on the force platform (9).

During the experiment, athletes warmed up based on their personal routines for 45–60 min. After warming up, markers were applied, and athletes stood in the anatomical position with both feet on the force platform for static data collection. This was followed by three times of 110 m hurdling tests, with a rest period of at least 10 min between each trial. This study selected the best performance with the shortest time from each athlete's test data for analysis. After each test, the athletes checked the conditions of the markers to ensure data integrity. A successful trial was defined as one in which participants did not adjust their steps before sprinting into the data collection area, and no markers were lost before and during the hurdle within the area. (Figures 1, 2).

**Figure 1:**
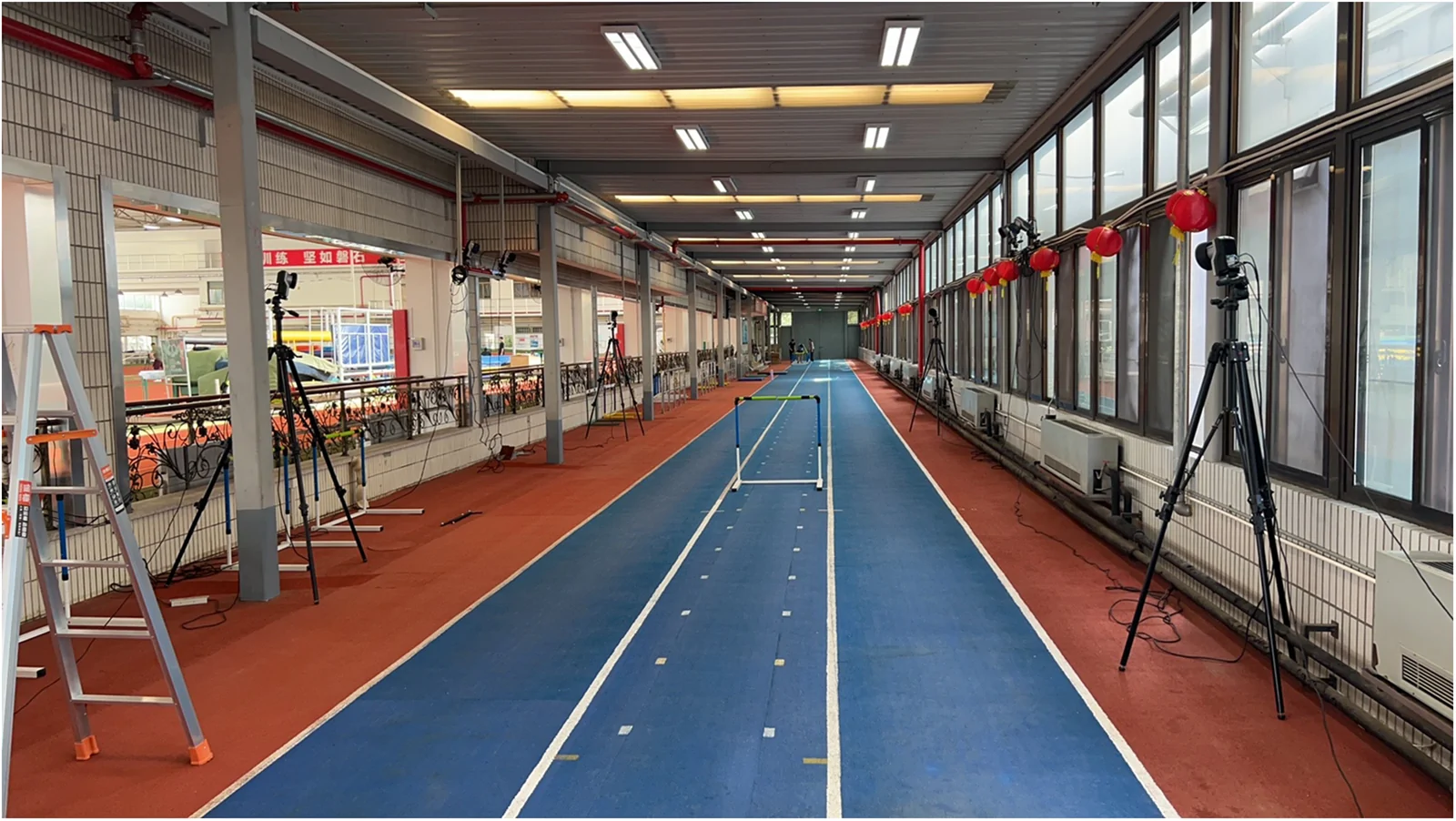
Experiment layout.

**Figure 2:**
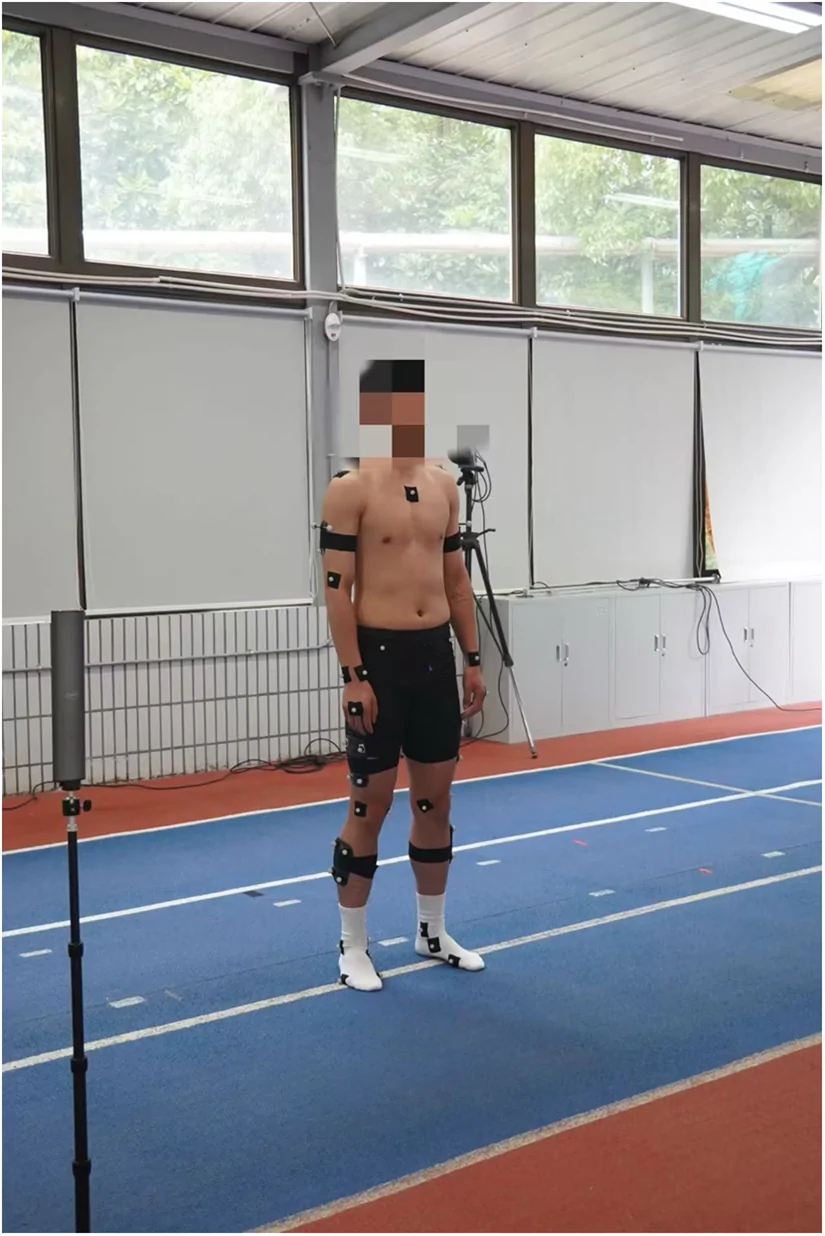
Mark points labeling plan.

### Data processing

Kinematic data were collected using QTM software and synchronized with the Kistler 3D force platform for dynamic data acquisition. The data were exported in C3D format and imported into Visual 3D (C-Motion, USA) to create a human model. The raw kinematic data and GRFs were processed using a Butterworth low-pass filter with a cutoff frequency of 10 Hz (28, 29). Net joint moments of the hip, knee, and ankle in the sagittal plane during the take-off phase were calculated using inverse kinetic. According to the Cardan sequence, hip flexion was defined as positive and extension as negative; knee flexion was defined as negative and extension as positive; ankle dorsiflexion was defined as positive and plantarflexion as negative. Joint moments and joint stiffness were normalized to body weight.

In this study, the take-off phase was defined as the moment when the foot first contacted the ground until the toe-off. The landing phase was defined as the interval from initial foot contact to the moment of maximum knee flexion, whereas the propulsive phase was defined as the interval from maximum knee flexion to toe-off (5) (Figure 3).

**Figure 3:**
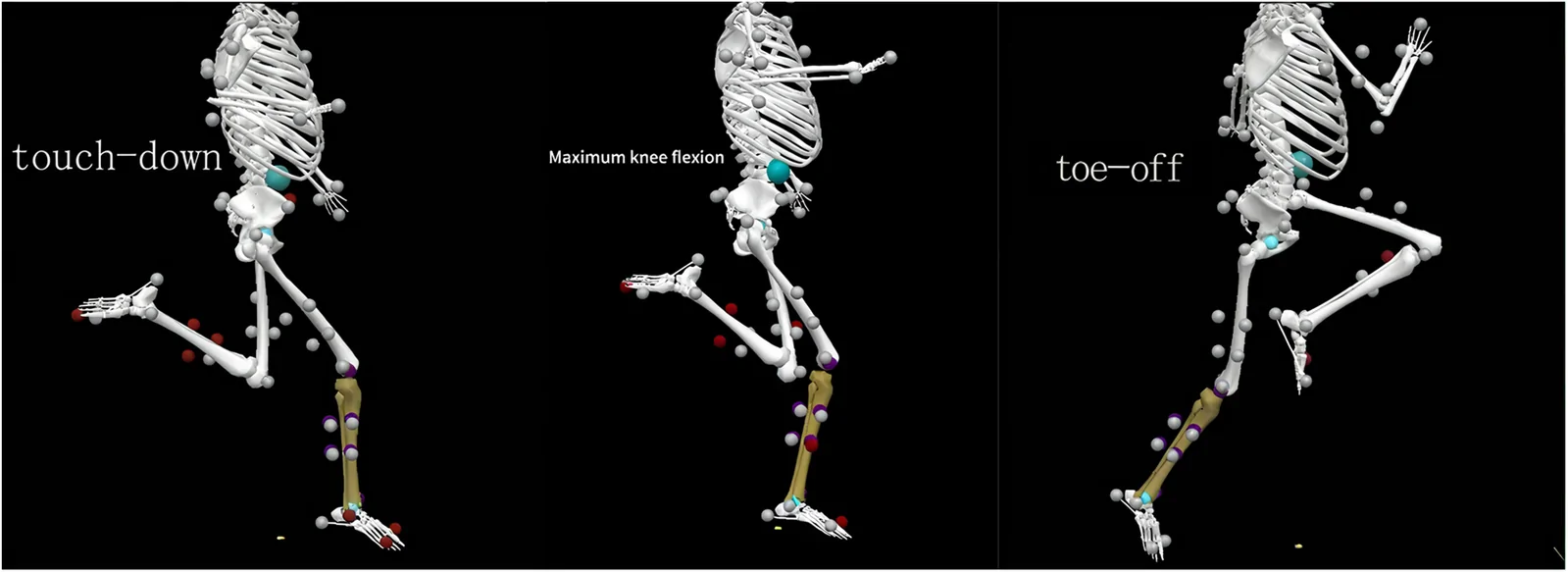
Phases division of take-off.

The formula for calculating joint stiffness is (19):

$$K_{\mathrm{jo}\mathrm{int}}=\frac{\Delta M}{\Delta \theta }$$

Δ*M* represents the change in lower limb joint moments, whereas Δ*θ* represents the change in lower limb joint angles.

The rate of change of velocity was calculated using the following equation:

$$v_{\mathrm{Loss}}=\frac{v_{\mathrm{TD}}-v_{\mathrm{TO}}}{v_{\mathrm{TD}}}\times 100\text{\%}$$

*v*_TO_, *v*_TD_ denote the horizontal velocities at take-off and touchdown, respectively.

### Statistical analysis

Statistical analyses were performed using IBM SPSS Statistics 27.0 (IBM Corporation, Armonk, NY, USA), with a significance level set at *P* < 0.05. Data are presented as mean ± standard deviation (*M* *±* *SD*). The Shapiro‒Wilk test was used to assess the normality of kinematic and dynamic variables. For normally distributed data, an independent sample t-test was used to compare the differences between the two athlete groups. Effect size (Cohen's *d*) and its 95% confidence interval were also calculated. *d* > 0.2 indicates a small effect, *d* > 0.5 indicates a medium effect, and *d* > 0.8 indicates a large effect. Pearson's bivariate correlation analysis was applied to examine the relationships between the relative peak moments and stiffness of the three lower-limb joints with velocity and rate of velocity change. The correlation levels were defined as follows: *r* = 0.1–0.3, low; *r* = 0.3–0.5, moderate; *r* = 0.5–0.7, high; *r* = 0.7–0.9, very high; and *r* = 0.9–1.0, extremely high (31).

## Result

### Peak joint moments of the lower limbs during the landing phase of take-off

During the landing phase of take-off, the peak hip joint moment was “+,” it indicating a hip flexion moment. The peak hip flexion moment for Group B(6.58 ± 0.94 Nm/kg) was significantly higher than that for Group A (4.69 ± 1.51 Nm/kg) (*P* < 0.01, Cohen's *d* = 1.03), Moreover, the relative peak hip joint moment was significantly positively correlated with touchdown velocity (*r* = 0.59, *P* < 0.05); During the landing phase of take-off, the peak knee joint moment was “−,” it indicating a knee flexion moment. The peak knee flexion moment for Group B (−2.54 ± 0.93 Nm/kg) was significantly higher than that for Group A (−1.38 ± 0.23 Nm/kg) (*P* < 0.01, Cohen's *d* = 0.44), and it was significantly positively correlated with initial down velocity. (*r* = 0.51, *P* < 0.05); During the landing phase of take-off, the peak ankle joint moment was “+,” it indicating a ankle dorsiflexion moment. The peak ankle moment for Group B (0.17 ± 0.04 Nm/kg) was significantly higher than that for Group A (0.11 ± 0.01 Nm/kg) (*P* *<* *0.01*, Cohen's *d* = 0.02), and it showed a significantly positive correlation with touchdown velocity (*r* = 0.61, *P* < 0.05). Moreover, the regression equations constructed between touchdown velocity and the relative peak moments of the hip, knee, and ankle joints during the braking phase were all statistically significant (Figures 4, 5, Table 2).

**Figure 4:**
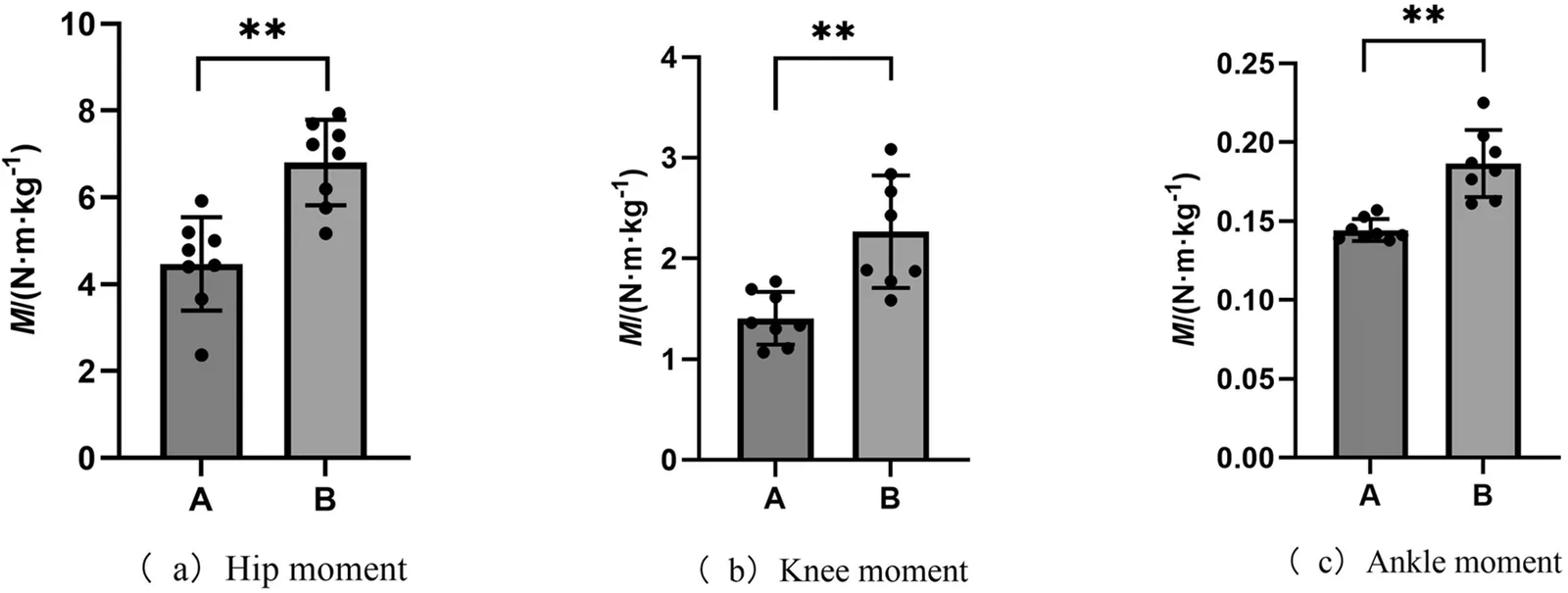
Relative peak joint moments during the landing phase. **(a)**, **(b)** and **(c)** represent the peak joint moment of the hip, knee, and ankle joints, respectively.

**Figure 5:**
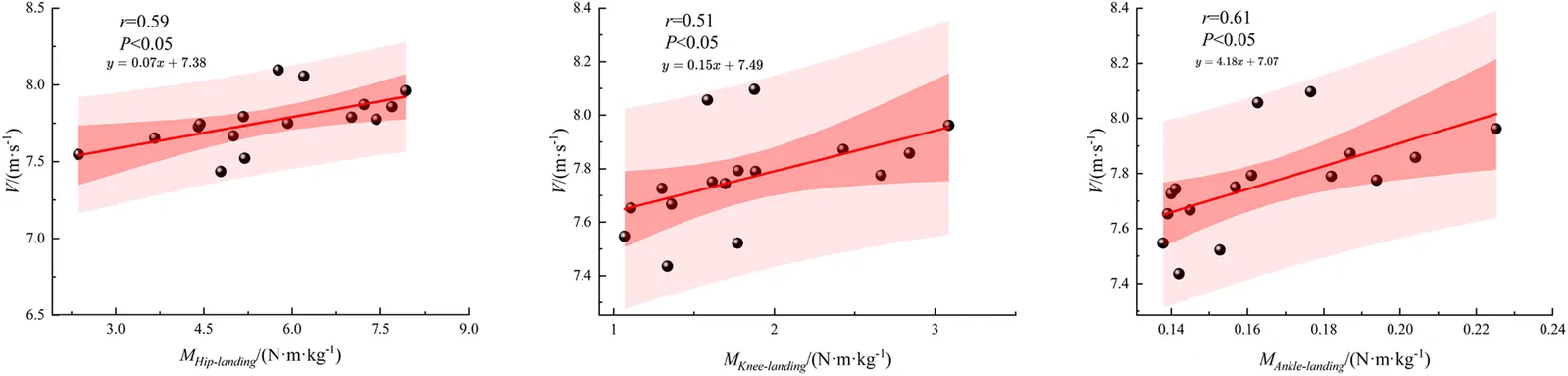
Correlation between touchdown velocity and relative peak moments of lower joints during the landing phase.

**Table 2:** Biomechanics data of lower limb joints in the take-off phase.

Indicator	Group A	Group B
	(*n* = 8)	(*n* = 8)
*v*_TO_	7.26 ± 0.38	7.80 ± 0.11
*v*_TD_	7.63 ± 0.12	7.90 ± 0.12
*M*_Hip−landing_	4.69 ± 1.51	6.58 ± 0.94*
*M*_Knee−landing_	−1.38 ± 0.23	−2.54 ± 0.93*
*M*_Ankle−landing_	0.11 ± 0.01	0.17 ± 0.04*
*M*_Hip−propulsive_	−3.81 ± 0.97	−8.67 ± 2.20**
*M*_Knee−propulsive_	1.29 ± 0.16	2.10 ± 0.44*
*M*_Ankle−propulsive_	−0.12 ± 0.01	−0.19 ± 0.04*
*K*_Hip_	0.15 ± 0.05	0.39 ± 0.19**
*K*_Knee_	0.13 ± 0.01	0.18 ± 0.11*
*K*_Ankle_	0.02 ± 0.01	0.12 ± 0.07**

*v*_TO_, *v*_TD_ represent the horizontal velocities at take-off and touchdown, respectively, expressed in m/s.; *M*_Hip−landing_, *M*_Knee−landing_, *M*_Ankle−landing_ denote the relative peak moments of the hip, knee, and ankle joints, respectively, during the braking phase of take-off.; *M*_Hip−propulsive_, *M*_Knee–propulsive_, *M*_Ankle–propulsive_ denote the relative peak moments of the hip, knee, and ankle joints, respectively, during the propulsive phase of take-off, expressed in N·m/kg.; *K*_Hip_, *K*_Knee_, *K*_Ankle_ represent the relative stiffness of the hip, knee, and ankle joints, respectively, expressed in N·m·[(°)·kg]^−1^.

* *P* < 0.01.

** *P* < 0.001.

### Relative peak joint moments of the lower limbs during the propulsive phase of take-off

During the propulsive phase of take-off, the peak hip joint moment was “−,” indicating a hip extension moment. The peak hip extension moment for Group B (−8.67 ± 2.20 Nm/kg) was significantly greater than that for Group A (−3.82 ± 0.97 Nm/kg) (*P* < 0.001; Cohen's *d* = 1.70). The peak knee joint moment during the take-off phase was positive, indicating a knee extension moment. The peak knee extension moment for Group B (2.09 ± 0.47 Nm/kg) was higher than that for Group A (1.31 ± 0.20 Nm/kg) (*P* = 0.002; Cohen's *d* = 0.36). The peak ankle joint moment during the take-off phase was negative, indicating a plantarflexion moment. The peak plantarflexion moment for Group B (−0.21 ± 0.02 Nm/kg) was greater than that for Group A (−0.16 ± 0.02 Nm/kg) (*P* < 0.001; Cohen's *d* = 0.02). These results indicate that higher-level athletes have a greater ability to generate joint moments during the take-off phase (Figures 6, 7, Table 2).

**Figure 6:**
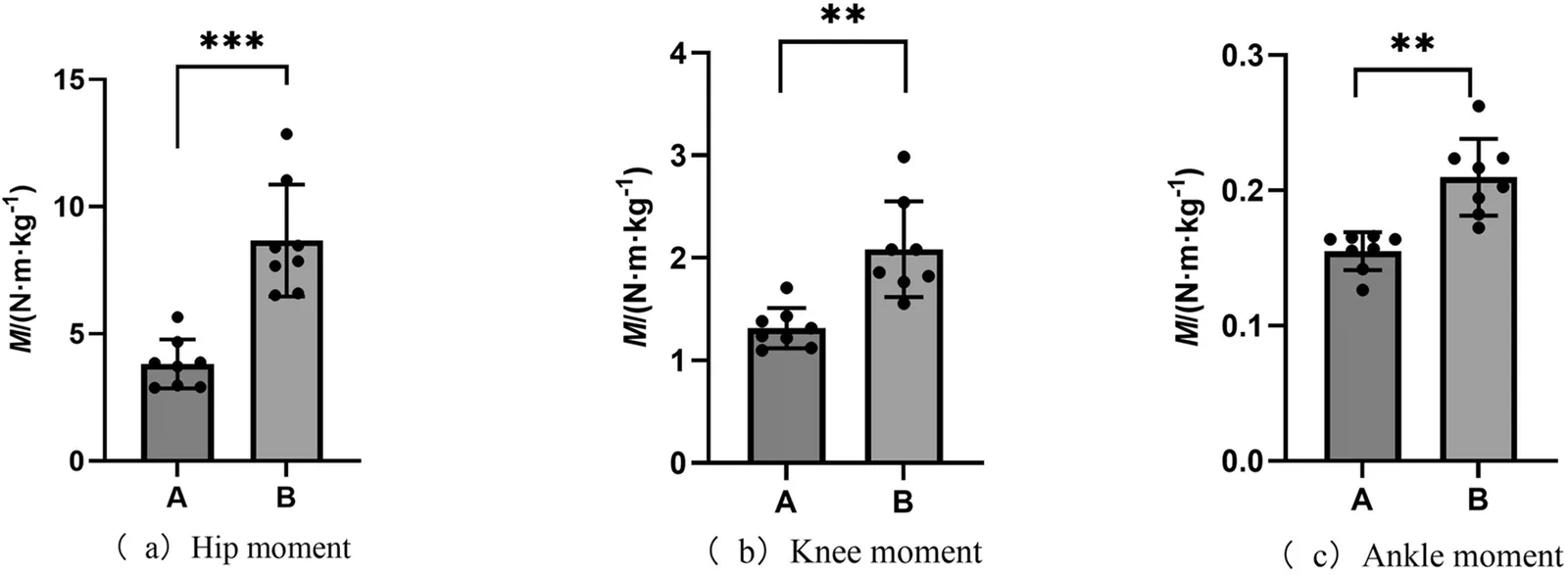
Relative peak joint moments during the propulsive phase. **(a)**, **(b)** and **(c)** represent the peak joint moment of the hip, knee, and ankle joints, respectively.

**Figure 7:**
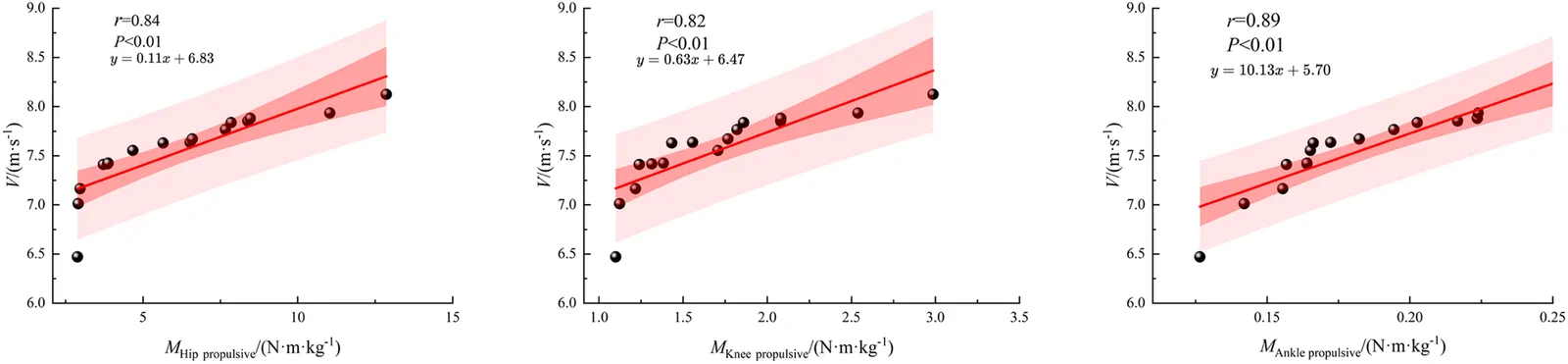
Correlation between take-off velocity and relative peak moments of the lower limbs during the propulsive phase.

### Joint stiffness

During the take-off phase, the hip joint stiffness for Group B (0.51 ± 0.12 Nm °/kg) was significantly higher than that for Group A (0.24 ± 0.02 Nm·deg^−1^·kg^−1^) (*P* < 0.001; Cohen's *d* = 0.09). Similarly, knee joint stiffness for Group B (0.39 ± 0.10 Nm·deg^−1^·kg^−1^) was significantly higher than that for Group A (0.22 ± 0.04 Nm·deg^−1^·kg^−1^) (*P* < 0.01; Cohen's *d* = 0.08). Ankle joint stiffness was also significantly higher for Group B (0.06 ± 0.01 Nm·deg^−1^·kg^−1^) than for Group A (0.04 ± 0.01 Nm·deg^−1^·kg^−1^) (*P* < 0.001; Cohen's *d* = 0.01). Hip, knee, and ankle joint stiffness exhibited highly negative correlations with the rate of change in toe-off velocity, with *r* values of −0.88, −0.90, and −0.95, respectively, *P* < 0.01. These findings suggest that athletes with higher performance levels have greater lower limb joint stiffness, indicating a stronger ability to resist joint deformation (Figures 8–10).

**Figure 8:**
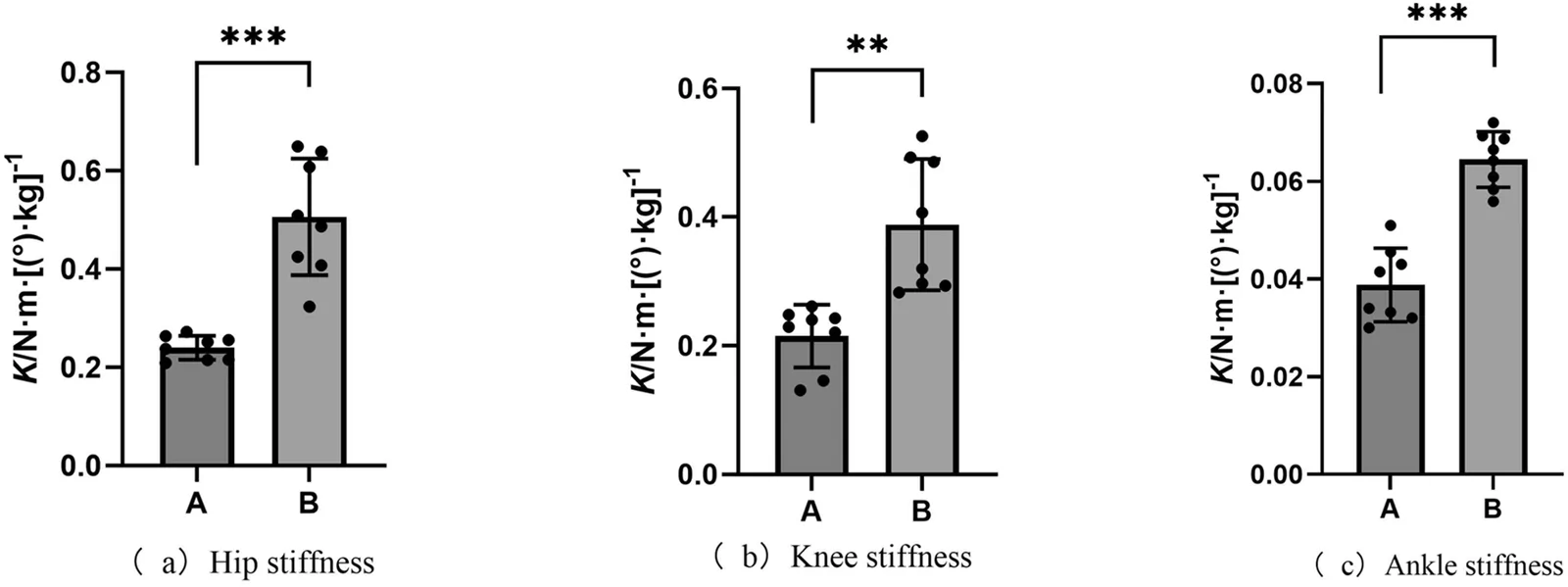
Comparison joint stiffness. **(a)**, **(b)** and **(c)** represent the peak joint moment of the hip, knee, and ankle joints, respectively.

**Figure 9:**
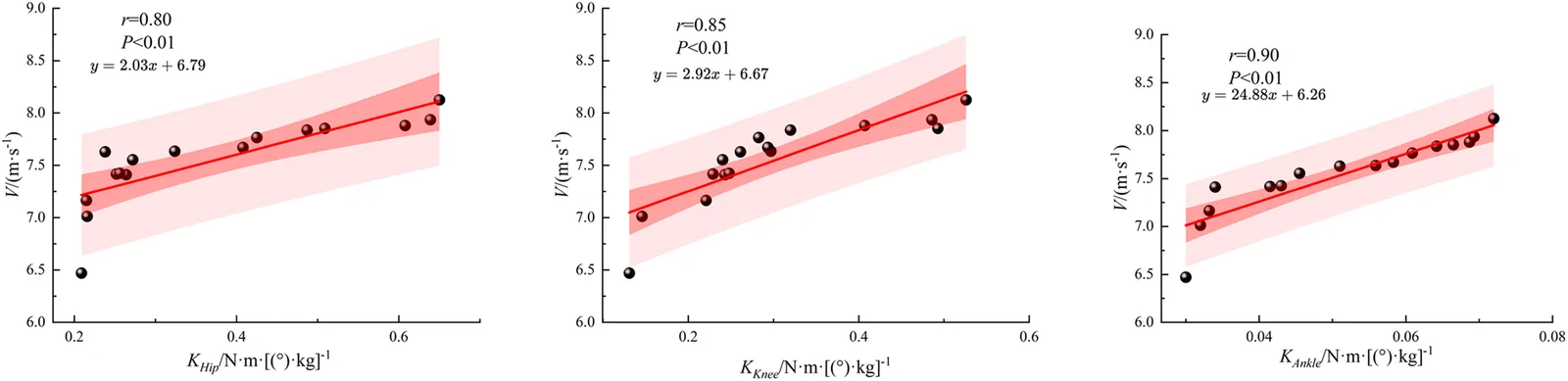
Correlation between joint stiffness and take-off velocity.

**Figure 10:**
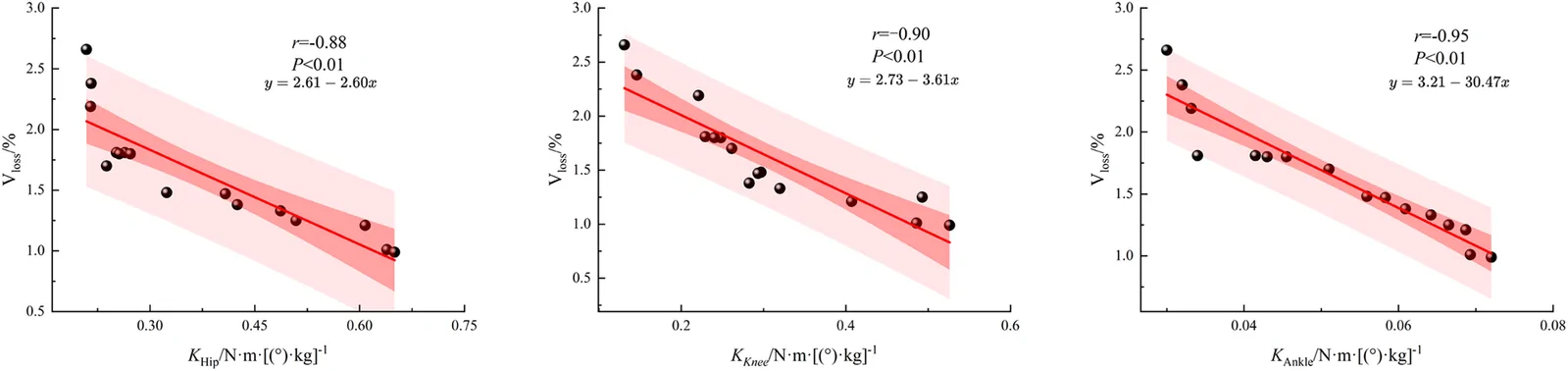
Correlation between velocity loss rate during the take-off phase.

## Discussion

### Peak joint moment

Relative peak joint moment is considered an important indicator for evaluating the mechanical load borne by joints during movement. In terms of hip joint peak moment, the present study found statistically significant differences between Group B and Group A in both the landing and propulsive phases of take-off. At touchdown, the hip joint experiences a substantial relative peak flexion moment, which is significantly positively correlated with touchdown velocity. During the propulsive phase, an increase in hip extension moment was accompanied by an increase in toe-off velocity. This finding is consistent with previous studies, which have highlighted the critical role of enhanced hip extensors and flexors in contributing to the propulsive force of the lower limbs during running (16). In the sprint, emphasis is placed on hip and pelvic musculature as the primary contributors to force production. Active hip extension during the support phase helps maintain a high center of mass and preserve horizontal propulsive forces. The involvement of the hip extensors in the early support phase has been demonstrated to be a key determinant of sprint performance (32). The hip extensor muscles play a dominant role during the support phase of running (13, 33). In hurdling, athletes must rapidly execute hip flexion followed by extension to successfully clear the hurdle. Unlike traditional techniques, modern sprint-hurdling emphasizes force production from the hip and pelvic musculature, recruiting lower-limb muscles to enhance propulsive while strengthening core engagement to improve upper–lower limb coordination and ensure greater stability during clearance the hurdle. This mechanistic difference largely explains why Group B exhibited a substantially higher peak hip extension moment than Group A. During the landing phase, the hip extensors undergo passive centrifugal contraction to store elastic energy, whereas in the propulsive phase they actively extend to drive the body forward rapidly and increase horizontal velocity, because hurdlers must generate a vertical force component during the take-off phase to elevate the center of mass and clear the hurdle, the range of hip extension throughout this phase is relatively limited. Moreover, differences in push-off angles between sprinting and hurdling lead to distinct hip joint movement patterns. In hurdling, the effectiveness of hip joint force production directly affects movement amplitude and velocity, making it a key factor in technical execution. Therefore, training should emphasize the development of explosive strength in the hip extensors to enhance movement speed, while also strengthening the hip flexors to maintain balanced musculature between flexors and extensors, thereby reducing the risk of hip joint injury.

With respect to knee joint relative peak moment, the present study found that Group B exhibited higher values than Group A in both the landing and propulsive phases of take-off, and these values were significantly correlated with toe-off velocity. In hurdling, the primary role of the knee joint during the support phase of take-off is to maintain the height of the body's center of mass and to transmit the force generated by the hip musculature to the ankle joint, an increase in horizontal velocity requires greater knee extension moment (16, 34). Mann et al. (32) reported that the normalized knee extension moment during high-speed running was 0.25 N·m/kg. The values observed in the present study were relatively higher, primarily because hurdlers must generate greater vertical and horizontal velocities during take-off to successfully clear hurdles with a height of 1.067 m. During the landing phase, the knee joint absorbs a greater amount of energy, which facilitates improved coordination between the ankle and hip joints (35). Increasing the relative peak knee joint moment during running can facilitate breakthroughs in maximal velocity. A faster horizontal velocity at touchdown effectively enhances the peak knee extension moment, the knee joint plays a particularly crucial role during the landing phase (13). With proper technique, improving knee joint moment not only helps maintain high running speeds but also reduces the risk of injury. Therefore, coaches should prioritize strengthening the knee extensors and enhancing lower-leg strength in hurdlers (25).

With respect to ankle joint relative peak moment, the present study found significant differences between Group B and Group A in both the landing and propulsive phases, and the relative peak moment was likewise significantly correlated with velocity. The ankle joint actively contributes to landing during the take-off phase, where the triceps surae store elastic potential energy eccentrically during braking and subsequently release it during propulsion phase. By minimizing breaking at touchdown and maintaining forward momentum, athletes can transition more rapidly into the propulsive phase. During the propulsive phase, the ankle joint quickly shifts from dorsiflexion at landing to plantarflexion, providing the kinetic energy necessary to clear the hurdle. The ankle plantarflexion moment is the primary source of forward propulsion during the propulsive phase. A greater plantarflexion moment can effectively facilitate ankle plantarflexion and shorten ground contact time. Schache et al. (13) reported that the ankle plantarflexion moment during maximal sprinting was 0.39 N·m/kg. The values observed in the present study were comparatively lower than those in sprinting (horizontal velocity: 8.95 m/s), which may be attributed to differences in horizontal velocity and movement patterns. In hurdling, athletes must maximize horizontal velocity while simultaneously generating sufficient vertical velocity during take-off to successfully clear the 1.067-m hurdle. This requirement is likely the primary factor contributing to the observed differences.

### Joint stiffness

The present study found a significant difference in hip joint stiffness between the two groups of hurdlers during the take-off phase, with hip stiffness showing a strong positive correlation with take-off velocity. Joint stiffness refers to the capacity of a specific joint to resist displacement, and it depends on both the type of movement performed and the mechanical characteristics involved in that movement (36). Greater joint stiffness is generally advantageous for running and jumping events (18). Jin et al. (35) examined joint stiffness across different running speeds and found that hip stiffness increased with velocity in both walking and running conditions, showing a strong positive correlation. This reflects an adaptive change in stiffness in response to velocity. In hurdling, this helps effectively maintain dynamic balance while providing stability and support for the body. Hip joint stiffness is determined by the skeletal structure and the synergistic action of the gluteal and iliopsoas muscles, and it governs the stability of single-leg support. During take-off phase, the hip joint must maintain an appropriate level of stiffness to balance explosive force production with joint loading. The hip joint serves as the primary source of driving force from touchdown to the end of the landing phase (14), the propulsive efficiency of the take-off leg depends on hip joint stiffness, and insufficient hip stiffness may result in inadequate extension during take-off phase, leading to hurdle collisions. The present study found that elite hurdlers primarily performed hip extension during the take-off phase, aiming to minimize horizontal velocity loss at touchdown and to increase horizontal velocity during the propulsive phase. During the take-off phase, the hip joint requires high stiffness to support the body, restrict hip joint range of motion, and shorten braking duration. This facilitates rapid and full extension of the take-off leg with effective hip drive, thereby enhancing the scissoring speed of the legs.

The present study also revealed a significant difference in knee joint stiffness between Group B and Group A, with knee stiffness showing a strong positive correlation with toe-off velocity. During running, knee joint stiffness plays a more critical role in controlling the leg compared with ankle joint stiffness (20). Greater knee joint stiffness can reduce velocity loss during the take-off phase and more effectively transmit the action of the hip extension moment to the distal segments, thereby propelling the body forward. During the take-off phase, the degree of knee flexion during landing directly influences the effectiveness of propulsion. Excessive knee flexion increases the load on the lower-limb muscles and prolongs ground contact time throughout the take-off phase. By increasing knee joint stiffness and reducing the extent of knee flexion, athletes can effectively shorten the duration of propulsion, thereby ensuring a smoother and faster clearance of the hurdle. During the sprint, increasing the range of knee extension can effectively reduce the load on skeletal muscles while maintaining a uniform distribution of joint forces and preserving the elastic properties of the joint (37). Research suggests that knee joint stiffness is also associated with the activation levels of the vastus medialis and rectus femoris. The nervous system regulates knee joint stiffness during the eccentric phase by modulating the activation of the flexor and extensor muscles, thereby reducing foot–ground contact time (38, 39). Therefore, training that enhances the strength of the vastus medialis and rectus femoris, along with improving the neuromuscular recruitment capacity, can help increase knee joint stiffness and shorten both the landing and propulsive phases of take-off.

The results of this study demonstrated a significant difference in ankle joint stiffness between Group B and Group A. Ankle stiffness was strongly positively correlated with toe-off velocity, and its correlation with the rate of velocity change was the highest among all lower-limb joints. This indicates that ankle stiffness can effectively minimize velocity loss during the take-off phase, thereby enabling a faster completion of the hurdle step. Belli et al. (15) suggested that during the sprint, the ankle joint functions to generate greater stiffness, shorten ground contact time, and propel the body forward. Modern 110 m hurdling requires athletes to maintain a high center of mass, fast stride frequency, and elevated take-off. The ankle joint must perform a rapid whipping action to actively “pull against the ground,” thereby generating the initial horizontal take-off velocity and an appropriate take-off angle. Greater ankle joint stiffness enables more effective utilization of stored elastic potential energy, allowing the joint's elastic properties to propel the body forward, shorten ground contact time, and improve mechanical efficiency. In hurdling, which requires repeated high-intensity support and propulsion, inadequate ankle stiffness increases the likelihood of various sports injuries. Therefore, routine training should incorporate stretch–shortening cycle exercises to enhance ankle stiffness.

This study primarily reveals the technical performance differences of athletes during the take-off phase through the analysis of peak joint moments and joint stiffness. Several studies have demonstrated that peak joint moments and joint stiffness play a critical role in reflecting an athlete's technical proficiency (40–42). High-level athletes typically exhibit higher peak joint moments and joint stiffness, which indicates that top performers are able to generate more force in a shorter time while maintaining better control over their movements, in contrast to lower-level athletes. The biomechanical characteristics of different movements are reliable indicators of athletic performance. However, other studies have suggested that time-series analysis of kinematic and dynamic data can more effectively capture the variations in technical execution throughout the movement cycle, optimizing the entire motion sequence (43). Therefore, future research should address the limitation of not incorporating time-series analysis of movement patterns, to further enhance the credibility of the findings.

## Conclusion

Compared with Group A, hurdlers in Group B exhibited higher peak moments and joint stiffness in the hip, knee, and ankle during the take-off support phase. Moreover, both peak joint moments and joint stiffness during take-off showed strong correlations with toe-off velocity. The hip extension moment and ankle plantarflexion moment contributed the most to velocity generation during the take-off phase, while the knee joint played a critical role in force transmission and coordination. Higher joint stiffness was associated with reduced velocity loss during take-off phase, with ankle stiffness showing the strongest correlation with the rate of velocity change among the three joints. The regression equations derived in this study can effectively predict the quantitative relationships between joint kinetic indicators and take-off velocity. This study clearly revealed the differences in take-off phase mechanics between hurdlers and demonstrated the close association between kinetic and kinematic parameters. Based on hurdling movement patterns, it is recommended that routine training incorporate stretch–shortening cycle exercises to enhance lower-limb strength and joint stiffness, with particular emphasis on the gluteus maximus, biceps femoris, and triceps surae.

## Limitation

Although this study provides valuable insights into hurdling technique, it still has certain limitations. First, the sample size in this study is relatively small, and the results may be individualized, lacking generalizability. Secondly, the study focused only on the joint dynamics during the take-off phase, whereas the landing phase and the three-step approach between hurdles are also critical. The technique during landing and the three-step approach between hurdles are key factors that determine hurdle performance. Lastly, this study was limited to the fourth hurdle. Future research should involve a larger and more diverse sample, including athletes of varying levels. Additionally, more comprehensive kinematic and dynamic data collection throughout the entire hurdling process is necessary to explore their impact on performance and velocity-related metrics.

## Data Availability

The original contributions presented in the study are included in the article/Supplementary Material, further inquiries can be directed to the corresponding authors.
